# Angelo Calli

**Published:** 1915-02

**Authors:** 


**Angelo Calli** died in Monza, November 3, aged 57. He was Professor of Hygiene in the University of Rome and had done notable work in regard to cholera, epidemic cerebro-spinal meningitis and the artificial cultivation of amoebae. He had been a member of the national parliament since 1892.

